# Predicting Vasovagal Responses: A Model-Based and Machine Learning Approach

**DOI:** 10.3389/fneur.2021.631409

**Published:** 2021-03-10

**Authors:** Theodore Raphan, Sergei B. Yakushin

**Affiliations:** ^1^Department of Computer and Information Science, Institute for Neural and Intelligent Systems, Brooklyn College of CUNY, Brooklyn, NY, United States; ^2^Graduate Center of CUNY, New York, NY, United States; ^3^Department of Neurology, Icahn School of Medicine at Mount Sinai Hospital, New York, NY, United States

**Keywords:** rat, vasovagal syncope, relaxation oscillator, baroreflex sensitivity, machine learning, modeling and simulation, vasovagal response

## Abstract

Vasovagal syncope (**VVS**) or neurogenically induced fainting has resulted in falls, fractures, and death. Methods to deal with **VVS** are to use implanted pacemakers or beta blockers. These are often ineffective because the underlying changes in the cardiovascular system that lead to the syncope are incompletely understood and diagnosis of frequent occurrences of **VVS** is still based on history and a tilt test, in which subjects are passively tilted from a supine position to 20° from the spatial vertical (to a 70° position) on the tilt table and maintained in that orientation for 10–15 min. Recently, is has been shown that vasovagal responses (**VVRs**), which are characterized by transient drops in blood pressure (**BP**), heart rate (**HR**), and increased amplitude of low frequency oscillations in **BP** can be induced by sinusoidal galvanic vestibular stimulation (sGVS) and were similar to the low frequency oscillations that presaged **VVS** in humans. This transient drop in **BP** and **HR** of 25 mmHg and 25 beats per minute (bpm), respectively, were considered to be a **VVR**. Similar thresholds have been used to identify **VVR's** in human studies as well. However, this arbitrary threshold of identifying a **VVR** does not give a clear understanding of the identifying features of a VVR nor what triggers a **VVR**. In this study, we utilized our model of **VVR** generation together with a machine learning approach to learn a separating hyperplane between normal and **VVR** patterns. This methodology is proposed as a technique for more broadly identifying the features that trigger a **VVR**. If a similar feature identification could be associated with **VVRs** in humans, it potentially could be utilized to identify onset of a **VVS**, i.e, fainting, in real time.

## Introduction

A neurogenically induced vasodilation and corresponding inappropriate bradycardia can lead to hypotension and transient loss of body tone and consciousness, that is, fainting. This condition has been termed neurogenic or vasovagal syncope (**VVS**) (1–4). Many nerves connect to the heart and blood vessels, which help control the beat frequency of the heart and the dilation and contraction of the blood vessels that control blood pressure (**BP**). Usually, these control signals are coordinated so that a decrease in **BP** is compensated by an increase in heart rate (**HR**) so that proper blood flow to the brain is maintained through sympathetic outflow (5). If the nerve signals are not coordinated, it could create a condition where the blood vessels dilate while the heart rate slows causing blood to pool in the legs and not enough reaches the brain, resulting in syncope (5). Although **VVS** is not considered harmful because lying down causes a resumption of blood flow to the brain, it can result in falls, fractures, and in some cases death (1, 2). Thus, a correct diagnosis of frequent occurrences of **VVS** is critical for the management of this disease. Yet, diagnosis of **VVS** is usually based on history and a tilt test, in which subjects are passively tilted from a supine position to 20° from the spatial vertical (to a 70° position) on a tilt table and maintained in that orientation for 10–15 min. This maneuver usually brings on the symptoms of fainting and returning subjects to a reclining position makes them go away. Treatment of recurrent **VVS** using implanted pacemakers or beta blockers is often ineffective (6–10), because the underlying changes in the cardiovascular system that lead to the syncope are still incompletely understood (11, 12), although there has been an acknowledgment that the relative timing of **BP** and **HR** plays an important role in **VVS** generation (3, 4). The role of the vestibular system in generating the timing has only recently been explored and has suggested ways of habituating **VVS** as a treatment option (13).

The interest in better managing **VVS** has seen a concerted attempt to relate **VVS** to drops in blood pressure (**BP**) and heart rate (**HR**), which have been termed vasovagal responses (**VVR's**). There have been suggestions that **BP** oscillations during tilt testing are a predictive marker for **VVS** (14). In support of this idea, multiresolution analysis with wavelets demonstrated that there was increased power in low frequency modulations of BP that presage an episode of **VVS** in a human fainter (15). Recently, **VVRs** have been generated in anesthetized rats by repetitively activating the Vestibulo-Sympathetic Reflex (**VSR**), using sinusoidal galvanic vestibular stimulation (**sGVS**) or with 70° head-up tilts and ±70° oscillation in pitch (16–18). Although in anesthetized rats there is no concept of fainting, the **VVRs** are surprisingly similar to those in humans and we postulated that they would be a good animal model for **VVRs** in humans and their corresponding **VVS** responses (16).

In the studies on the rat, vasovagal oscillations were induced by sinusoidal galvanic vestibular stimulation (sGVS) with low frequency oscillations (16–18) in each of six rats. These low frequency modulations in **BP** and **HR** were referred to as vasovagal oscillations (18). In some instances, **sGVS** induced a substantial fall in BP and HR, that is, the transient **VVR**, which recovered over several minutes. We identified this transient component as a **VVR**, if there was a drop in BP and HR of 25 mmHg and 25 beats per minute (bpm), respectively. Similar thresholds have been used to identify **VVR's** in human studies as well (14, 15). These data have shown that **VVRs** may be an outcome of an aberrant type of vestibular stimulation of the vestibulosympathetic reflex and not a disease (17). The data also show that the rat may be a useful animal model for understanding how human **VVRs** may be generated and studied. However, this arbitrary threshold of identifying a **VVR** from **BP** drops does not give a clear understanding of the identifying features of a **VVR** nor what triggers a vasovagal response. The purpose of this study is to utilize our model of **VVR** generation (19) together with a machine learning approach to identify a separating hyperplane (20) between a normal and **VVR**, based on simultaneous **BP** and **HR** changes. This technique would more broadly identify the features that trigger **VVRs**. If a similar feature identification could be associated with **VVR**s in humans, it potentially could be utilized to identify **VVS** onset of fainting in real time.

## Methods

### Experimental Methods

Adult, male Long-Evan rats (Harlan Laboratories, MA) weighing 300–400 g were used in these studies. All experiments were approved by the Institutional Care and Use Committee of the Mount Sinai School of Medicine. In this study, the data were taken from previous studies (16, 17, 21). We give a brief summary of the surgical and **sGVS** procedures.

### Surgical Procedures

The implantation of a blood pressure measurement device and a head fixation mount were accomplished during the same aseptic surgical session. Throughout the surgery, rats were kept on a heating-pad controlled by the feedback of a rectal temperature probe. The surgery and testing were conducted under isoflurane anesthesia (4% induction, 2% maintenance).

### Implantation of Bolts to Allow Painless Fixation of the Head During Experiments

Bolts were secured with dental acrylic cement, and two nuts were encased semisoft acrylic. A telemetric blood pressure sensor (DSI, St Paul, MN) was implanted in the abdominal aorta. These animals were utilized in a series of experiments performed over the next 2 months [see (16) for details].

### Sinusoidal Galvanic Vestibular Stimulation for Inducing VVR

During testing, the heads of the rats were immobilized using the head mounts attached to a cylindrical holder for the animal's body. Sinusoidal currents generated by a computer-controlled stimulator (22) were delivered via two Ag/AgCl needle electrodes inserted into the skin over the mastoids, behind the external auditory meati. sGVS was given binaurally with currents of 1–4 mA and frequencies of 0.008 to 0.5 Hz. Current and frequencies were randomized, and 15–30 min were allowed to elapse between stimuli to reduce possible effects of habituation.

### Tilt Protocol to Initiate VVR

The rats were statically tilted 70° and held in this position until they developed a vasovagal response. If a vasovagal response developed or if there was no response after several minutes, they were then brought back to the prone position.

### Data Collection and Analysis

**BP** in response to sGVS was recorded continuously using customized A/D conversion hardware (Grass Technologies, West Warwick, RI) and Polyview software (Grass technologies) and stored at a rate of 1 KHz. BP data from the telemetric sensors were collected via a wand receiver (DSI, St Paul, MN) at 1 KHz with 12 bit resolution (Data Translation, Inc.) using our data collection program. The data were converted for analysis into what we have referred to as Virtual Memory File (VMF) format. The data format is comprised of channels that represent stimuli and responses and can be representations of analog data, that are acquired via an A/D converter or event channels that associate an event with a time of occurrence. The VMF application software that we developed contains modules, which operate on the data and perform a wide range of transformations, such as a correlation analysis, power spectral analysis, timing of events, etc. The program also has visualization capabilities so that data can be displayed as time functions. The transformed data can also be displayed in the frequency domain or as one variable against another [See (23) for a more thorough description].

**BP** was utilized to obtain heart rate (**HR**) off-line. HR was identified from the peaks in BP. Stored pulses were converted to instantaneous frequency (beats·s^−1^) and stored in a separate channel for further analysis. Mean square sinusoidal fits to the data were used to estimate variations of **BP** and **HR** to the sinusoidal oscillations.

## Results

### Data Underpinnings of Modeling Vasovagal Responses

**Figure 1:**
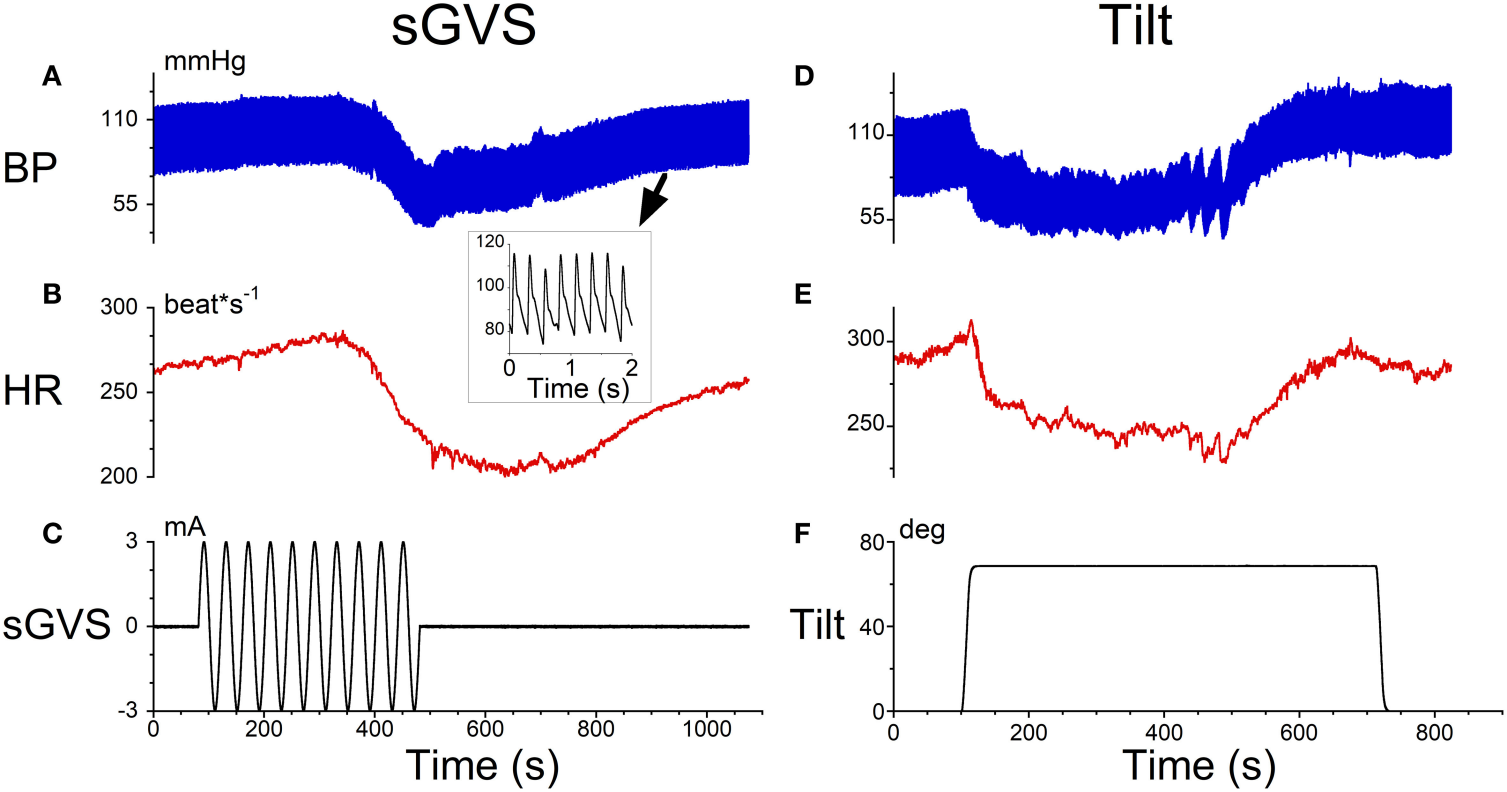
Typical data that initiated a **VVR** in an anesthetized rat obtained from sGVS **(A–C)** and head up tilts of 70°. The inset in **(A)** shows a 2 s time scale. There were triangular type oscillations corresponding to the rapid transition (systole) and the slower transitions (diastole). **(A)** At a 1,000 s time scale, there was a coordinated drop in the in **BP** oscillations **(A)** as well as in **HR (B)** during sGVS **(C)**. Similar drops in **BP (D)** and **HR (E)** occurred during head up tilts of 70° **(F)**.

There were two characteristics of typical data that characterized a **VVR** during sGVS and tilts in anesthetized rats that were used to model the response (Figure 1). At a 2 s time scale, there were triangular type oscillations corresponding to the rapid transition (systole) and the slower transitions (diastole) (Figure 1A, inset). At a 1,000 s time scale, there was a coordinated drop in the in **BP** oscillations (Figure 1A) as well as in **HR** (Figure 1B) during sGVS (Figure 1C). Similar kinds of drops in **BP** (Figure 1C) and **HR** (Figure 1E) occurred during head up tilts of 70° (Figure 1F). The responses of **BP** and **HR** are similar to that observed during head up tilts when testing for fainting (15) and strategies for stopping **VVS** by leg-crossing and muscle tensing (24). The model therefore encompassed the systolic-diastolic oscillations and a central control structure to predict the drops in **BP** and **HR** over the longer time scale (19). The modeling approach in this paper considers what features of the model can be used to better identify the drops in **BP** and **HR** using the data and machine learning.

### Modeling Approach

**Figure 2:**
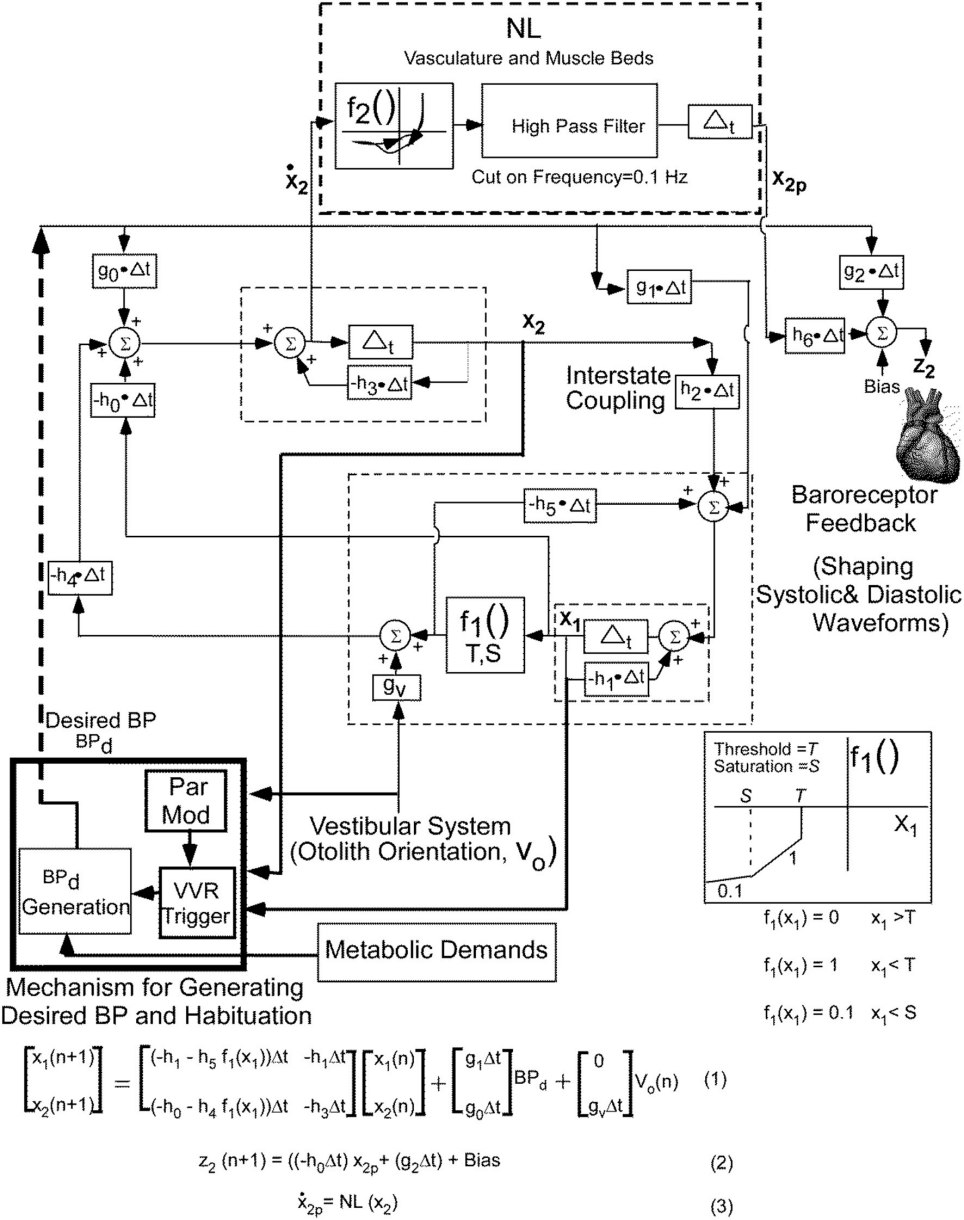
Non-linear relaxation oscillator model that generates the systoles and diastoles seen in the blood pressure signal together with the central mechanisms that control the **BP** and **HR** variations and adaptations. See text for details. This figure has been modified and extended from (19).

The basis of the model (Figure 2) that generates the triangular systolic-diastolic oscillations is a relaxation oscillator (19, 25). There is a signal Desired Blood Pressure (**BP**_**d**_), which acts as input that maintains the relaxation oscillations and is dependent on metabolic needs of the muscles and cells (26). This model not only simulates the basic diastolic and systolic behavior of **BP** as monitored by an intra-aortic sensor, but also predicts their variations in response to vestibular stimuli. The model also showed that alterations in **BP**_**d**_ changed the oscillation amplitude and its frequency, which compared favorably with data on systolic **BP**, pulse pressure, and Baroreflex Sensitivity **(BRS**) (19). It also predicts that as the **BP** drops, the period of the systolic-diastolic oscillations increases, and therefore **HR** drops (19). It is this relationship that is explored in this paper to elucidate the features that best describe a **VVR** and by extrapolation to predict a **VVS**.

Briefly, the relaxation oscillator model, which is of second order, has two states, **x**_**1**_ and **x**_**2**_, which are generated by delays Δ_t_ with feedback elements that are discrete representations of leaky integrators (dashed rectangles) in continuous system models (27). The non-linear element (**f**_**1**_, Figure 1), was modeled as piecewise linear with threshold, **T**, and saturation, **S**. This nonlinear feedback is responsible for making the system oscillate, mimics the non-linear feedback present in the baroreflex (28, 29) and is a minimal structure for inducing relaxation oscillations (25). The realization of these integrators by central circuits is not known, but may be accomplished through functional commissural connections between neurons in the rostral ventrolateral medulla (RVLM) and those in the nucleus tractus solitarius NTS) (30, 31). The vestibular system affects the oscillator in two ways: First, by adding an input in the feedback loop from the otolith orientation signal **V**_**0**_ through **g**_**v**_. Second it affects the parameter modification of the **VVR** trigger, which causes a drop in **Bp**_**d**_, initiating a **VVR** (Figure 2).

In order to fit the data better on both systole-diastole transition and its derivative, a non-linear function (**NL**, **f**_**2**_**)** of the derivative of the integrator state (**x**_**2**_) was implemented as an enhancement of the model developed in Raphan et al. (19) (Figure 2). The output of this non-linearity was processed by a high pass filter with a cut-in frequency of 0.1 Hz and then processed by an integrator, whose output has been labeled **x**_**2p**_. We postulate that this output combined with **BP**_**d**_ through parameters **h**_**6**_ and **g**_**2**_, which is offset by a bias (Bias) generates the signal **z**_**2**_ that controls **BP** and presumably the Volumetric Flow rate by constricting and dilating the muscle beds (32, 33). The purpose of the high pass filter was to prevent drifts at the output of the integrator due to any level component (**DC**) generated by the non-linearity, **f**_**2**_. The oscillator maintained the systolic to diastolic transitions and the non-linearity shaped and constrained the **BP** and its derivative, which fit the data. The equations below the model (Figure 2) were implemented in Matlab and used to simulate model predictions.

### Model Predictions of Data and Defining the Features for Identifying a VVR

**Figure 3:**
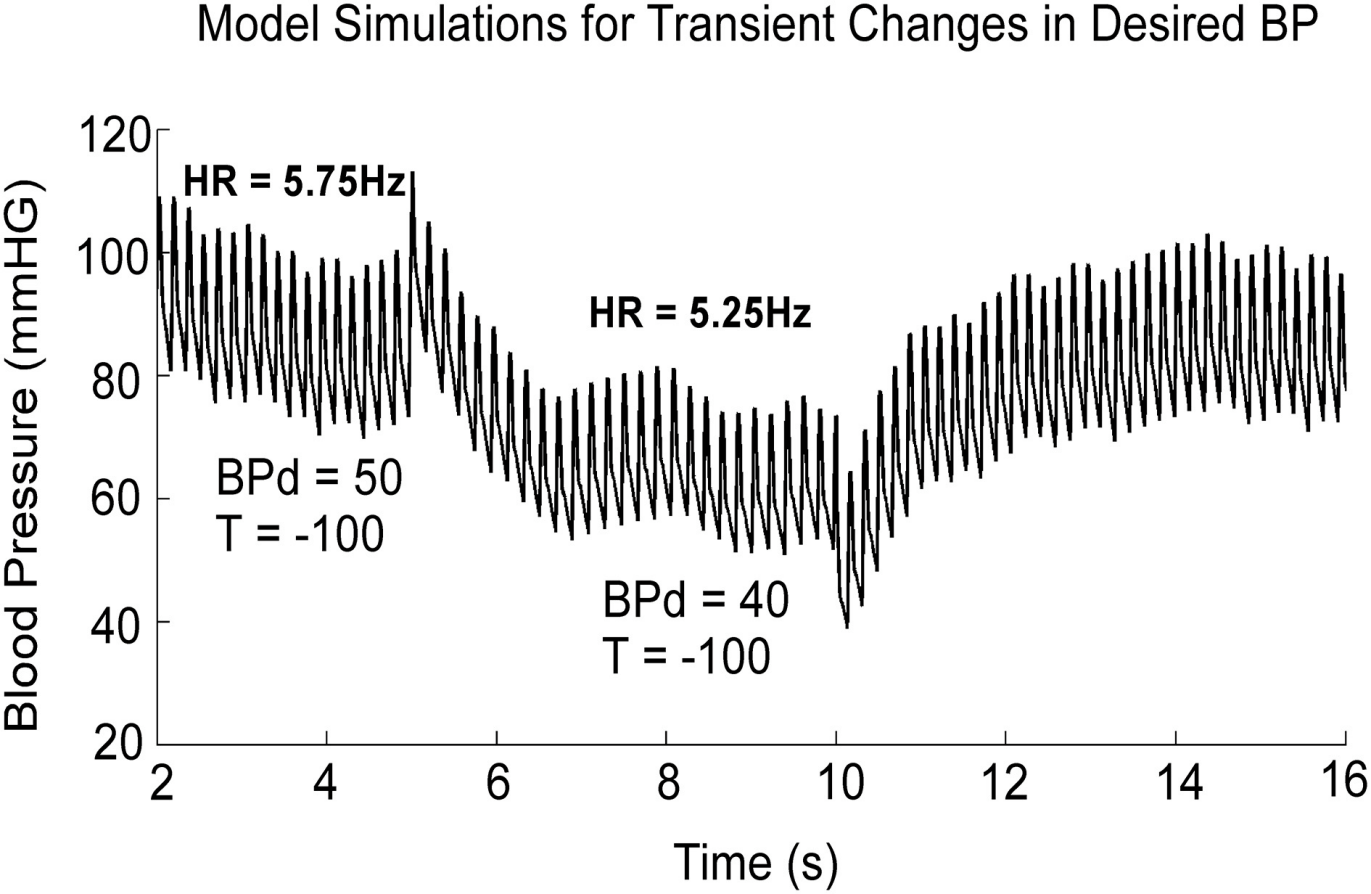
Model simulation showing the effects of dropping **BP**_**d**_ from 50 to 40 mmHG. The threshold, **T**, in the feedback loop, responsible for the transition from diastole to systole was held constant at −100. **BP**_**d**_ is the only parameter that effectively mimics a **VVR** when it is dropped from its stable value. This figure was adapted from (19).

The simulations presented here are only those that have a direct bearing on the **VVR**. A more complete analysis of the model performance and the testing of the model against a wide range of data is given in (19). When activated by a constant **BP**_**d**_, the output of the model, **z**_**2**_, oscillated at a fixed frequency and the systolic and diastolic phases compared favorably with those from an anesthetized rat when there was no external vestibular stimulus (Figure 3, **BP**_**d**_ = 50). When **BP**_**d**_ was dropped at *t* = 5 s from 50 to 40 (Figure 3), due to the peak to peak amplitude of each simulated systole was reduced (Figure 3), and **BP** had the same properties as the experimental data (18, 19). Thus, the model had the flexibility to simulate experimental data not only in the normal state, but also during a **VVR**. Altering parameters, such as the threshold, or other parameters did not produce a **VVR** with these characteristics (19). Thus, a key prediction of the model is that it is a drop in **BP**_**d**_ that triggers a **VVR**.

From the above findings, we hypothesize that in non-susceptible animals, or in animals that are habituated, the internal signal, **BP**_**d**_, is prevented from dropping (13). We have also postulated that the **BP**_**d**_ signal originated in specific circuits that reconstruct this signal from states of the relaxation oscillator. We have further postulated that there is a specific trigger circuit that monitors the states and output variables of the relaxation oscillator in the brainstem as well as vestibular inputs to the baroreflex feedback, correlates these signals and determines whether to initiate a **VVR**. In order to determine the signals that activate this mechanism, we propose a new method for identifying **VVR**s, which utilizes a metric that combines features associated with both **BP** and **HR**.

**Figure 4:**
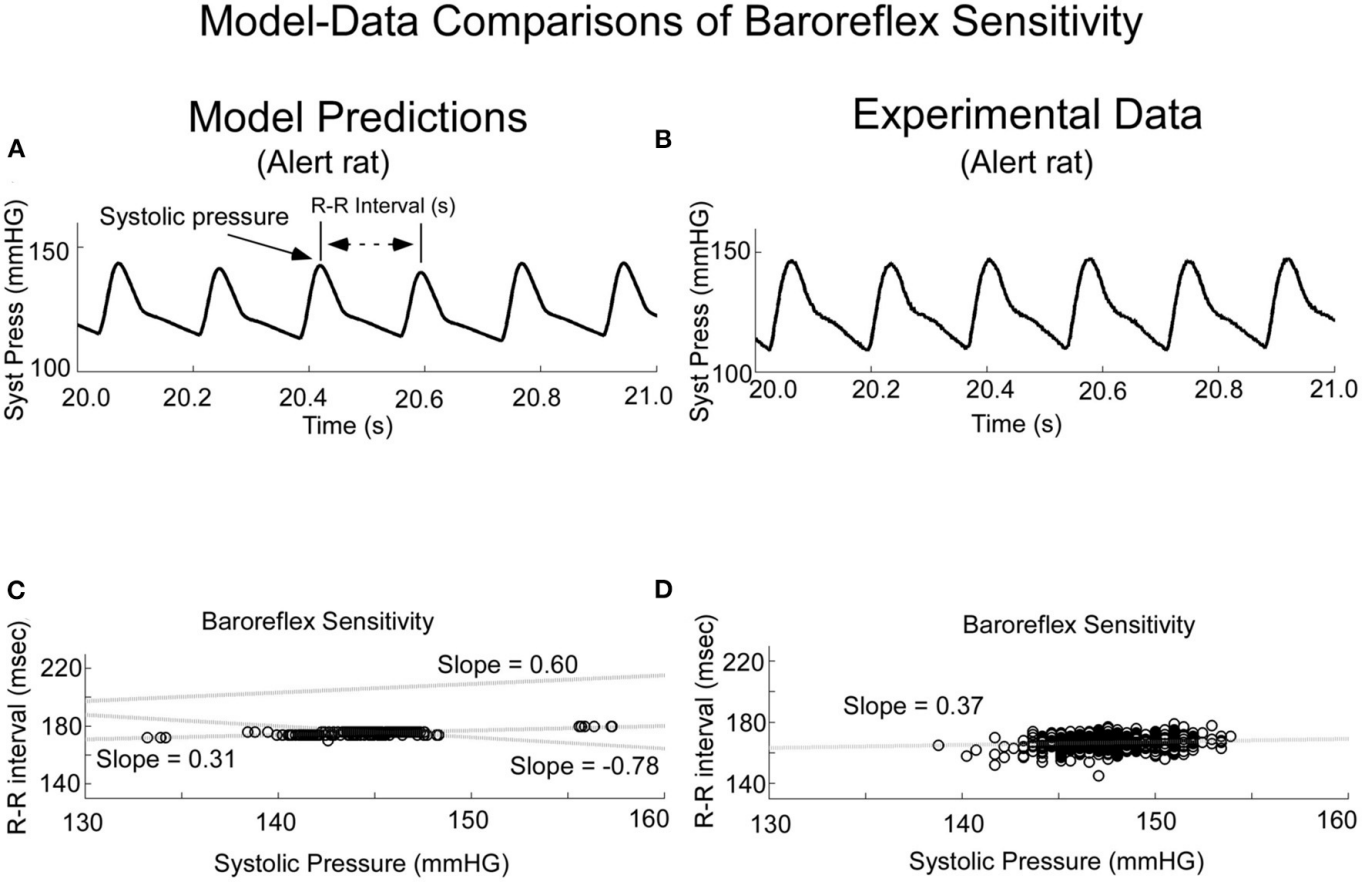
Comparison of Model Predictions **(A**,**C)** and Experimental Data **(B**,**D)** of Baroreceptor Sensitivity (BRS). The model predicted the experimental data **(A**,**B)** and had the flexibility to predict the Baroreflex Sensitivity **(C**,**D)**. This figure was taken from (19).

These features were derived as follows: we incorporated stochastic variations in threshold of the baroreflex feedback, **T**. When this was done, the model predicted what has commonly been referred to as baroreflex sensitivity (**BRS**) (Figure 4), which has been defined as the slope of the regression when R-R interval is plotted as a function of previous systolic pressure (34, 35). When the threshold, **T**, was varied randomly, the model output achieved varying systolic levels and intersystolic intervals (Figure 4A). This was the approximate behavior of the systolic levels and intersystolic intervals in the alert rat (Figure 4B). When the intersystolic interval was plotted as a function of Systolic Pressure **(BRS**), the model predicted a positive correlation for the **BRS** for vestibular input **V**_**0**_ =0 in the model (Figure 4C, Shaded dotted line, slope = 0.31). This compared favorably with the data obtained in the alert rat (Figure 4D) as well as habituated anesthetized rats (13). It also predicted the **BRS** of anesthetized rats (Figure 4C, slope = 0). For a constant input **V**_**0**_ = 10, the slope was increased to 0.6, which was the range of the baroreceptor in humans (34). The slopes could be altered by changes in sinusoidal vestibular input. (Figure 4C, slope −0.78), showing the wide range of baroreflex sensitivities that could be obtained from the model.

It has been noted that both **BP** and **HR** drops characterize a **VVR** (18, 36, 37). We have identified a signal **BP**_**d**_ that when it drops, both **BP** and the inverse of intersystolic interval drop. It is therefore of interest that baroreflex sensitivity is a parameter that is determined from a ratio of intersystolic interval and systolic **BP**. Thus, the **BRS** parameter has information about instantaneous **HR** and **BP**, rather than **BP** alone and could be the important trigger signal for **VVR** initiation. In this study, we therefore examined the temporal variations in **BRS** to determine the normal variation in anesthetized and alert states and the threshold that needs to be reached to generate a **VVR**.

**Figure 5:**
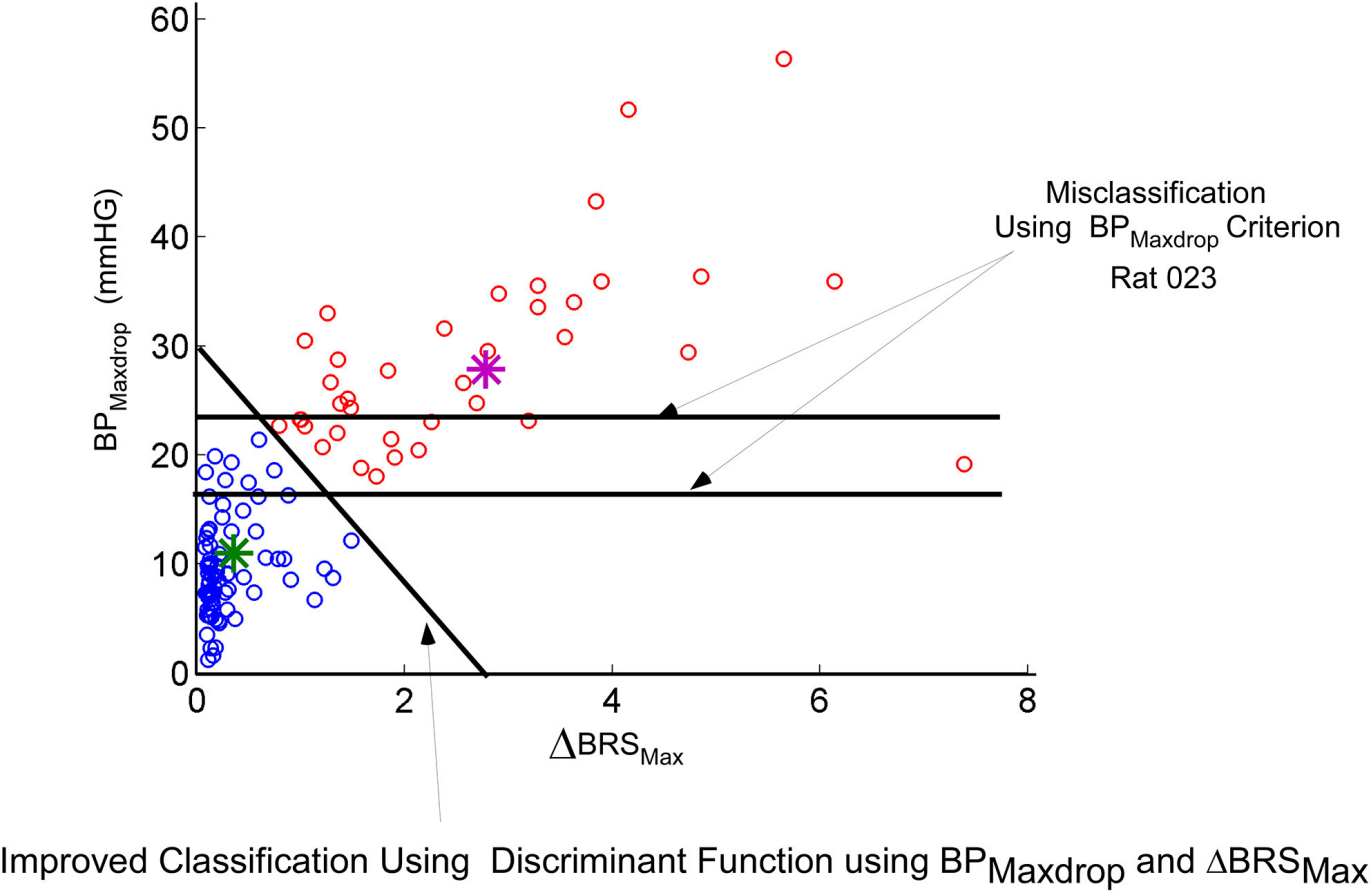
Machine learning approach to finding a linear discriminant function that seperates a known **VVR** from normal. The blue circles are not **VVR**s while the others are **VVR**s. The features that are being plotted are the maximum drop in **BP** (**BP**_**Maxdrop**_) vs. maximum change in the new metric Δ**BRS** (See Text). Using a threshold on **BP**_**Maxdrop**_ alone (horizontal lines) misclassifies a significant greater **VVR**s than does the learned linear discriminant function, which utilizes a combination of **BP**_**Maxdrop**_ and Δ**BRS**.

We then considered an instantaneous Baroreflex Sensitivity function (Δ**BRS**) to determine whether this feature is a better prognosticator of **VVR**s and determine how the states of the model are related to Δ**BRS**. The computation of Δ**BRS** was implemented as a moving average window of instantaneous ratios of changes in intersystolic interval (related to **HR**) to changes in systolic **BP**. When a **VVR** occurred, the peak drop in systolic **BP**, **BPmaxdrop**, was plotted vs. the maximum of Δ**BRS** (Δ**BRS**_**max**_). This plot had a considerable overlap in classifying a **VVR** according to a criterion based on maximal drops in **BP** alone (Figure 5). If the threshold for **BPmaxdrop** is chosen at 25 mmHG, then there are many **VVR**s that are missed (Figure 4, orange circles below 25 mmHG). If the threshold for classifying a **VVR** is 18 mmHG, many **non-VVRs** are classified as **VVR**s (Figure 5, blue circles). This shows that choosing a threshold for **BPMaxdrop** alone for identifying a **VVR** is an insufficient metric. A “**Machine Learning**” algorithm was used to find the discriminant function between what we termed **normal vs. VVR** (Figure 5 Separating Linear function of orange from blue circles). First a small “test set” of what had been identified as **VVR**s using large values of **BPmaxdrop** was clasiified (Figure 5, purple star). The remaining data points were classified using the learned discriminant function based on the test set. There was improved identification and separation of a **VVR** from a **non-VVR** (Figure 5). This shows that a linear combination of **BP**_**Maxdrop**_ and Δ**BRS** was a more appropriate metric.

**Figure 6:**
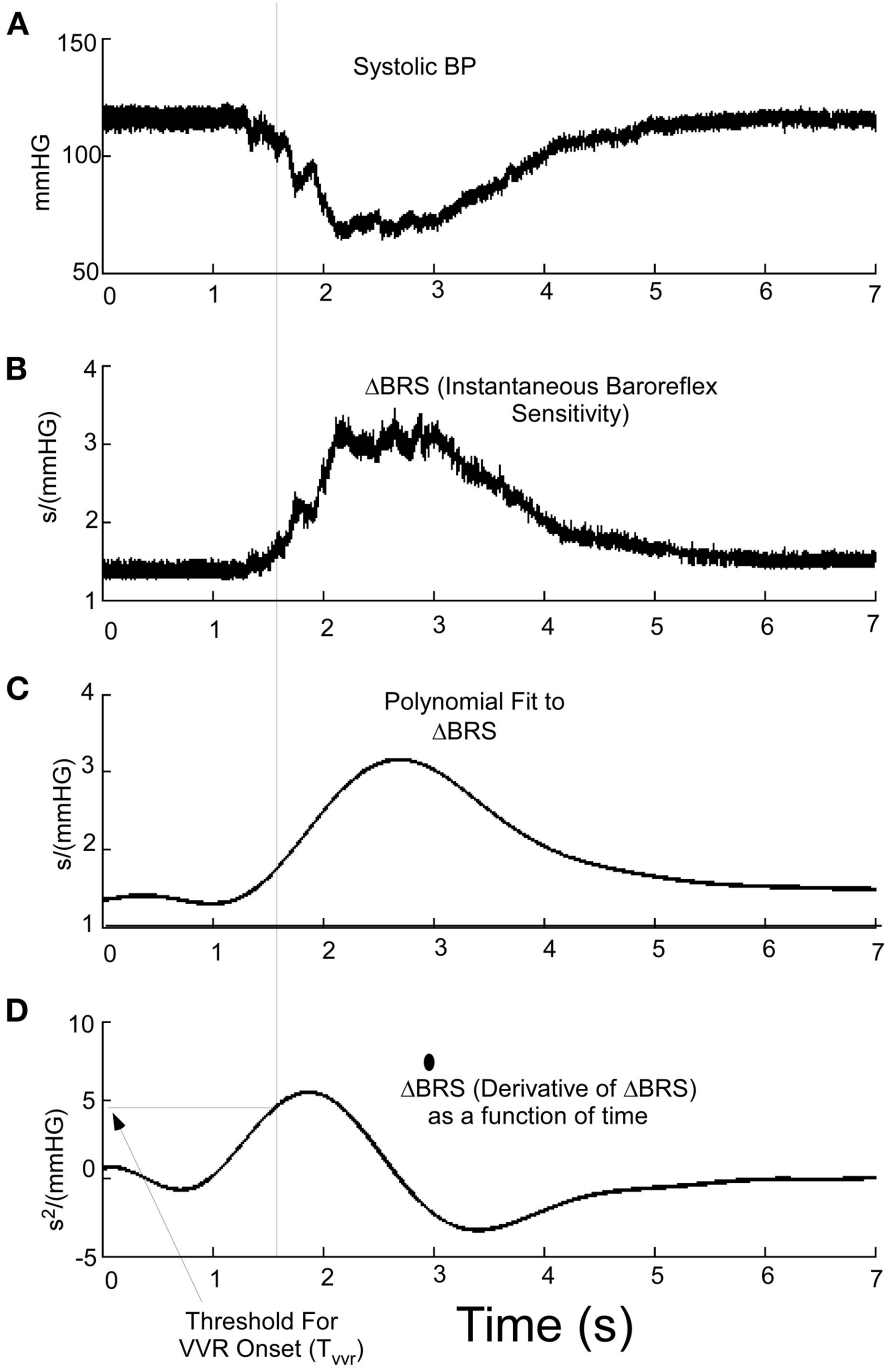
**(A)** Systolic **BP** as a function of time during a **VVR**. **(B)** Using the instantaneous baroreflex sensitivity, Δ**BRS**, allows for the polynomial fitting **(C)** and determining the derivative where a threshold (**T**_**vvr**_) **(D)** aligns and is a predictor of the drop in systolic pressure at the beginning of a VVR.

Because Δ**BRS** is an important part of classifying a **VVR**, we then considered whether the time series Δ**BRS** (Figure 6B) or its derivative (Figure 6D) could be used (Figure 6A) to predict the triggering of a **VVR**. The polynomial fit to Δ**BRS** (Figure 6C) is an intermediate step in finding the derivative and suggests that the derivative of Δ**BRS r**eaches a threshold, **Tvvr** (Figure 6D), before there is a drop in systolic level of **BP** (Figure 6, **Dotted Vertical Line**). This threshold metric, **Tvvr**, which is derived from the temporal characteristics of the derivative of Δ**BRS**, is consistent with the idea that Δ**BRS** is an important part of the discriminant function that separates a **VVR** from a non-**VVR**. Therefore, this newly defined Δ**BRS** function and its derivative are better predictors of a **VVR** and could be a metric that combines both **BP** and **HR** as determinants of an impending **VVR** and associated **VVS** in humans if a large data set were utilized.

## Discussion

This study has shown that this newly defined function, Δ**BRS**, and its derivative are predictive of a **VVR** and potentially could be important in predicting **VVS** in humans. This is consistent with the finding that a better discriminant for identifying a **VVR** is when **BP**_**max**_ is combined with Δ**BRS**_Max_. This new metric takes into account a critical feature of **VVR**s in humans and rats as the simultaneous occurrence of both bradycardia and hypotension at the onset of the **VVR** (38). It is supported by the idea that it is the loss of baroreflex function that triggers a **VVS** (17, 36, 39). Consistent with this, there is also an immediate loss of the baroreflex-generated Muscle Sympathetic Nerve Activity (MSNA) at the onset of syncope (40–42). The pathophysiological mechanism and significance of the baroreflex disengagement in producing bradycardia and hypotension have been conjectured to be the basis for **VVS** (3, 4), but no specific mechanism has been identified that could produce these changes. As such, despite being relatively common (1, 12, 43), the origin and neural basis of **VVR**s, which are related to **VVS** are not known (2, 36, 38) and there are no physical signs of neurogenically mediated **VVR**. Therefore, identifying the features that could be correlated with triggering the a **VVR** that underlies **VVS**, could be important in predicting their onset and managing the condition.

The pressure and flow dynamics are not easily modeled from a biophysical perspective. We have therefore taken a system theoretic approach to this problem to model the neural control of the cardiovascular oscillations as an internal model that entrains the natural cardiovascular oscillations (19). This approach encompasses not only the physical volumetric flow rate and pressure dynamics, but also how the **BP** and **HR** system is controlled by sensory-motor neural mechanisms that constrain the shape of the systolic-diastolic oscillatory waves (19). It had been suggested that the systolic-diastolic oscillations in **BP** had characteristics of a relaxation oscillator (44, 45). Recently, we demonstrated how relaxation oscillations could be embedded in a control system that could regulate its performance. In this model, the shape of the systolic/diastolic waveform is not determined strictly by the heart, but by a neural network, which mimics the oscillation features of the heart, referred to as the internal model. The feedback mechanisms implement closed loop control and drive actuators through non-linear mechanisms that can rapidly control the constriction of the arteries and vascular beds (33), regulating volumetric **blood flow rate** and **BP** (19). This kind of control is referred to as Model Reference Adaptive Control (**MRAC**) (46–48). The output of the internal model is compared to a feedback signal from baro-receptors that code **BP**, generating an error signal whose control parameters are updated based on this error signal. The control parameters then converge to ideal values that cause the actual **BP** and **HR** to ~match the response of the reference model. We have simulated the reference model behavior as a function of alteration of specific parameters and tested the predictions against data. This kind of control is different from eye movement control or leg control. These systems are stationary and need to be activated to have them move. The blood pressure and heart rate control requires the maintenance of oscillations by an already oscillating system. Therefore, it is reasonable that the control strategies would be different.

The model was implemented as a state determined second order relaxation oscillator, whose oscillation characteristics are governed essentially by a threshold and saturation mechanism in the feedback loop and a driving signal that maintains the oscillations. The threshold non-linearity, which has been observed in the baroreflex feedback (28, 29, 49), puts the system into systolic or diastolic mode. The model was adapted from work done on modeling oculomotor oscillations during nystagmus (25) and repetitive motion of the legs during locomotion (50, 51) where there are also oscillating fast and slow components of the stepping. An important enhancement to the model above over that presented previously (19), is a component, which we have labeled Mechanism for Generating **BP**_**d**_ and habituation. We propose that this component generates **BP**_**d**_ to maintain the relaxation oscillations. We also postulate that it correlates the signals arising from the states of the relaxation oscillator that is responsible for activating the **VVR** trigger, which inhibits **BP**_**d**_ Generation and initiates a **VVR**. Finally, it is responsible for activating the **Par Mod** component during habituation that adapts the parameters of **BP**_**d**_ Generation, which raises the threshold for triggering a **VVR** as habituation is ongoing (13).

There is some evidence that mechanism for generating **BP**_**d**_ and the parameter modification for maintaining **BP** oscillations or initiating a **VVR** is performed in the uvula. Optogenetic inhibition of Purkinje cell activity in the uvula modulates **BP** when anesthetized rats are tilted (52). There are also afferent (53) and efferent (54) connections to autonomic nuclei in the rabbit. These findings together with the fact that neural activity in the caudal medial, lateral and descending VN (55–58) are critical for **VSR** (59, 60) may contain a mediator that modulates **BP** (61–63) is consistent with the model structure that is being proposed.

In conclusion, much of the efforts in identifying the onset of a **VVR** has been focused on **BP** (14, 15, 18). The **VVR**s in rats were associated with increased power in the low frequency band (0.025–0.05 Hz) with synchronous oscillations in **BP** and **HR**, which have been termed vasovagal Oscillations; higher frequencies of **sGVS** rarely induced a **VVR** (18). The approach in this study was to base our criteria for predicting a **VVR** on a model that (1) predicted the approximate triangular shape of systolic/diastolic oscillations. (2) identified parameters, which could modify the oscillations. (3) Encompassed a theory for how amplitudes and frequencies of these systolic/diastolic oscillations could be controlled by vestibular input (4). Better Identified the features that could be used to separate a **VVR** from the normal systolic/diastolic oscillations using a machine learning technique.

The method developed was based on small data set, yet proved valuable in finding a separation between the systolic-diastolic beats during **VVR** and normal beats. Finding discriminant functions using a “Big Data” set for each animal should give us a better understanding of how **BP** and **HR** are processed to generate a **VVR** and how it is reflected in our newly defined Δ**BRS** function and its derivative. If a similar feature identification could be associated with **VVR**s in humans, it potentially could be utilized to identify onset of fainting in real time.

## Data Availability Statement

The raw data supporting the conclusions of this article will be made available by the authors, without undue reservation.

## Ethics Statement

The animal study was reviewed and approved by IACUC, Icahn School of Medicine at Mount Sinai Hospital.

## Author Contributions

TR contributed to the overall conceptual framework of this study. TR was responsible for developing the model, defining and testing the parameters that were used to identify **VVRs** and showing the plausibility of such an approach. TR also was responsible for the organization and writing of the manuscript. SY contributed by performing the experiments on the rats as well as contributing of the writing of the manuscript.

## Conflict of Interest

The authors declare that the research was conducted in the absence of any commercial or financial relationships that could be construed as a potential conflict of interest.
